# Participant and research team perspectives on the conduct of a remote therapeutic COVID-19 clinical trial: A mixed methods approach

**DOI:** 10.1017/cts.2022.397

**Published:** 2022-05-02

**Authors:** Denise H. Daudelin, Sarah K. Brewer, Alyssa B. Cabrera, Dorothy Dulko, Harry P. Selker

**Affiliations:** 1 Tufts Clinical and Translational Science Institute, Tufts University, Boston, MA, USA; 2 Institute for Clinical Research and Health Policy Studies, Tufts Medical Center, Boston, MA, USA

**Keywords:** Remote clinical trial, COVID-19, research participant experience

## Abstract

**Background:**

Responding to the need to investigate potential treatments of COVID-19, a research team employed a telehealth platform to determine whether niclosamide, an oral anthelmintic drug that had shown antiviral activity, reduced SARS-CoV-2 shedding and duration of symptoms in patients with mild-to-moderate symptoms of COVID-19. To encourage compliance with patient self-quarantine, this randomized placebo-controlled clinical trial was conducted utilizing a remote telehealth design to complete all study visits, monitor symptoms, and coordinate participant self-collected specimens.

**Methods:**

A mixed methods approach employing surveys and interviews of trial participants and interviews of research team members was used to collect their experiences with and perspectives on the acceptability of the remote clinical trial design and delivery.

**Results:**

Of the 67 eligible trial participants invited to take part in a study to evaluate the telehealth platform, 46% (*n* = 31) completed a post-participation survey. While 97% (*n* = 30) of respondents had not previously participated in a clinical trial, 77% (*n* = 24) reported they would consider taking part in a future remote research study. The majority of respondents were moderately or very comfortable (93%) with using the technology.

**Conclusions:**

The COVID-19 crisis was a call to action to expand understanding of the conduct of remote clinical trials, including the experiences of research participants. Our findings showed that this approach can be both effective for the conduct of research and positive for participants. Further research on the use of telehealth research platforms seems warranted in rural, underserved populations, and remote trials of prevention, screening, and treatment.

## Introduction

The emergence of COVID-19 led to many challenges throughout the world, one of which was the conduct of clinical research with patients while under quarantine. Telehealth, or the use of electronic information and telecommunication technologies to support remote clinical care, has been used for many years and had broader implementation during the COVID-19 pandemic [1], including for clinical trials. Telehealth use and conducting remote clinical trials require research teams to adapt study designs, embrace remote technologies, ensure sufficient participant recruitment and retention, and engage participants to complete trial activities.

Barriers to clinical trial participation result in low trial enrollment and failure to achieve recruitment and retention goals. Challenges to high enrollment rates include the lack of transportation and long distances to treatment locations with attendant financial costs of travel, parking, and lodging, the need to miss time from work and caregiving obligations, and the fatigue of frequent study and clinical care visits [2–4]. Failing to recruit sufficient numbers of participants carries significant costs, including wasted resources and time, and discouraged research staff, participants, and sponsors [5,6]. It also can contribute to non-generalizable samples due to bias in enrollment of those who can surmount hurdles and who are easily accessible to researchers. A promising approach to overcoming these barriers is the integration of technology such as telehealth visits, digital consent, and application of remote patient monitoring devices into clinical trial design [3,7–9]. In addition, for some studies, research participants collect their own samples at home and mail them to a lab for analysis [10,11]. Conducting research visits via telehealth mitigates travel-related time and expenses, can reduce wait times and missed appointments, and allows for greater scheduling flexibility [12].

Recent clinical research studies have assessed the feasibility of, and patient experiences with, remote technology. These studies have suggested that participants with various conditions, including type II diabetes, Parkinson’s disease, and heart failure, found telehealth visits and remote monitoring acceptable [13–16]. In addition, patient collection of nasal swab samples was shown to be feasible in a telemedicine study of COVID-19 [17]. Patients with COVID-19 enrolled in telehealth patient monitoring programs post-discharge reported high ratings of satisfaction with the quality and safety of their care and were less likely to be readmitted [18–20]. Less is known about the experiences of research participants with COVID-19 with respect to telehealth visit experience, self-reporting of symptoms and vital signs, and self-sample collection, and in the context of a pandemic where the understanding of the disease and its treatments were rapidly evolving, causing fear and uncertainty [17].

To determine whether niclosamide, an anthelmintic drug that has shown antiviral activity, reduces SARS-CoV-2 shedding and duration of symptoms in patients with mild-to-moderate COVID-19, a randomized double-blind placebo-controlled clinical trial was conducted [21]. The trial enrolled nonhospitalized individuals testing positive for SARS-CoV-2 by reverse transcriptase– polymerase chain reaction (RT-PCR) at Tufts Medical Center and the Tufts Medicine health system in Massachusetts from October 1, 2020, to April 20, 2021. Tufts Medical Center is located in Boston, MA, a diverse urban setting with a race ethnicity breakdown of 22% Black, non-Hispanic, 10% Asian, 8% White, Hispanic, 4% White, Multiracial Hispanic, and 45% White, non-Hispanic [22]. Clinical trial recruitment materials were translated into Simplified Chinese, Spanish, Portuguese, and Haitian Creole to support recruitment of non-English-speaking individuals.

Participants were asked to self-collect oropharyngeal and fecal samples for viral shedding testing at intervals during the trial. A Health Insurance Portability and Accountability Act (HIPAA) compliant telehealth platform was used to conduct all study visits remotely and to monitor participants’ self-report of symptoms and to assess adverse events at days 1-7, 10, 14, 21, and 30. Informed consent was obtained during a telehealth visit with a study physician and study team witness. A secure electronic signature was provided by the participant via DocuSign. A study kit, including the study drug (blinded niclosamide or placebo), a thermometer, study pill diary, pulse oximeter, instructions, and sample collection materials, was delivered via courier to all participants upon enrollment. Study activity instructions were reviewed by a study team member at each scheduled telehealth study visit. Study team contact information was provided to each participant for questions or concerns that arose between visits. Samples were self-collected by participants to prevent unnecessary hospital visits and to encourage compliance given the self-quarantine status of enrolled patients. Sample collection instructions were reviewed with participants, and oropharyngeal sample collection was directly observed by a research team member at each study visit. The samples were returned to a CLIA-certified lab via overnight delivery.

This article summarizes the results of a pre-specified evaluation of the trial participants’ and research team members’ perspectives and experiences with the remote conduct of the clinical trial and the likelihood of future remote trial participation. We sought to understand the barriers, facilitators, and benefits of remote trial conduct and to identify recommendations for future remote trials.

## Methods

We used a mixed methods approach employing surveys and interviews of trial participants and interviews of research team members to collect their experiences with and perspectives on the acceptability of the remote clinical trial design and delivery.

### Quantitative Survey

We developed an online trial participant survey to assess participant experiences with telehealth technologies by adapting existing validated survey instruments [23–26]. Additional questions about at-home sample collection, courier services, and interactions with research team members were included. We also collected information on whether trial participants would be willing to participate in a future clinical trial under various conditions (e.g., only attending study visits via a telehealth platform as opposed to in-person). The survey was pilot tested with five individuals representing different backgrounds and levels of familiarity with telehealth. Pilot testers were asked to read questions aloud and discuss their thought process as they answered the questions. The survey was revised to ensure that questions were written in plain language and were easily understood.

Once they had completed all study activities, trial participants were asked by the clinical trial research nurse or study coordinator if they would like to participate in the survey. Those who agreed were sent a HIPAA compliant email invitation, information sheet, and REDCap survey link. Chinese- and Spanish-speaking patients received translated surveys, and all survey participants were told that their participation was voluntary. Survey data were collected and managed using REDCap electronic data capture tools hosted at Tufts Medical Center [26,27].

In accordance with the inclusion criteria for the overarching trial, all participants in our study had COVID-19 as evidenced by PCR, were 18 years of age or older, experienced mild-to-moderate symptoms of COVID-19, and did not require hospitalization at the time of their enrollment in the trial. Recruitment of trial participants for the survey occurred between November 2020 and June 2021. The evaluation study was deemed exempt research by the Tufts Health Sciences Institutional Review Board.

### Qualitative Interviews

Separate stakeholder interview guides were developed for trial participants and trial research team members by three of the authors (DD, AC, SB). Training for interviews consisted of conducting a mock interview, with one author playing the role of a research participant, followed by feedback for the “interviewer.” Interviewers adhered to the script of the interview guide to ensure consistency, but were encouraged to ask probing questions in order to elicit thorough and clear responses. The goals of the interviews were to collect from trial participants and from research team members, their experiences and perspectives on the barriers and facilitators to using the telehealth platform for study visits, the ease or usefulness of at-home sample collection, interest in future participation in remote or in-person research, and recommendations for future improvements.

Additionally, for trial participants, the interview questions expanded on the survey questions in order to elicit detailed responses. Starting in April 2021, trial participants were recruited via email for a 30-minute interview. In order to reduce the possibility of recall bias, trial participants must have completed the trial on or after March 20, 2021. The decision to conduct participant interviews was made at the mid-point of niclosamide trial recruitment. As such, early niclosamide study participants were not invited to participate due to concerns related to recall bias. Trial participants received a $25 gift card for their time. Trial research staff were recruited for interviews in July 2021, after the trial was complete.

### Data Analysis

Survey responses were exported from REDCap into Excel for descriptive analyses, and survey questions were cross-tabulated with trial participant demographic data using SAS software (*SAS® Enterprise Guide® 8.3: User’s Guide 2020)* [29]. We looked for relationships between survey responses and demographic data, as well as relationships between participation in the survey and completion of key study activities (e.g., sample collection and telehealth visits).

Interviews with study participants were audio-recorded, with participants’ permission. The interviews were then transcribed for coding and analyses by two independent study staff who identified key quotes from the interviews. For niclosamide research team interviews, study staff took detailed notes on the interviews, independently organized their notes on interviewee responses into categories, and met to resolve differences through consensus, analyzing the content for emerging themes [30]. Interviews with niclosamide research staff were not recorded due to the intent to identify general themes rather than conduct in-depth coding and analysis. Study staff comfort with disclosing their experiences privately without concern of recorded responses was also considered.

## Results

### Quantitative Survey

Of the 67 trial participants invited to take part in the survey, 46% (*n* = 31) completed the survey. Table 1 compares demographic characteristics for those who completed the survey and those who declined. We also examined survey response rates based on the extent to which trial participants completed trial activities such as attending telehealth visits and completing sample collection.

**Table 1.** Demographic characteristics and completion of niclosamide trial activities for survey respondents and non-respondents

Characteristic		Non-respondents (*n* = 36)	Respondents (*n* = 31)
Age	Median	32	31
Ethnicity	Hispanic or Latino, % (*n*)	57% (4/7)	43% (3/7)
	Not Hispanic or Latino, % (*n*)	53% (32/60)	47% (28/60)
Sex	Female, % (*n*)	31% (8/26)	69% (18/26)
	Male, % (*n*)	68% (28/41)	32% (13/41)
Race	Asian	80% (4/5)	20% (1/5)
	Asian, White (Multiple)	0% (0/1)	100% (1/1)
	Black or African American	50% (2/4)	50% (2/4)
	Other	75% (3/4)	25% (1/4)
	White, % (*n*)	51% (27/53)	49% (26/53)
Preferred Language	Non-English	83% (5/6)	17% (1/6)
	English	51% (31/61)	49% (30/61)
Completed Telehealth Study visits	Completed all visits, % (*n*)	51% (29/57)	49% (28/57)
	Missed at least 1 visit, % (*n*)	70% (7/10)	30% (3/10)

Response rates differed by gender, with 69% (*n* = 18) of women participants completing the survey versus only 32% (*n* = 13) of men. There were notable differences in response rate by race, with 36% (*n* = 5) of non-white trial participants responding to the survey, versus 49% (*n* = 26) of white trial participants providing response data. Of those who missed at least one trial telehealth visit, 70% (*n* = 7) did not complete the survey. Statistical tests for significance were not performed due to small sample size.

**Fig. 1.**
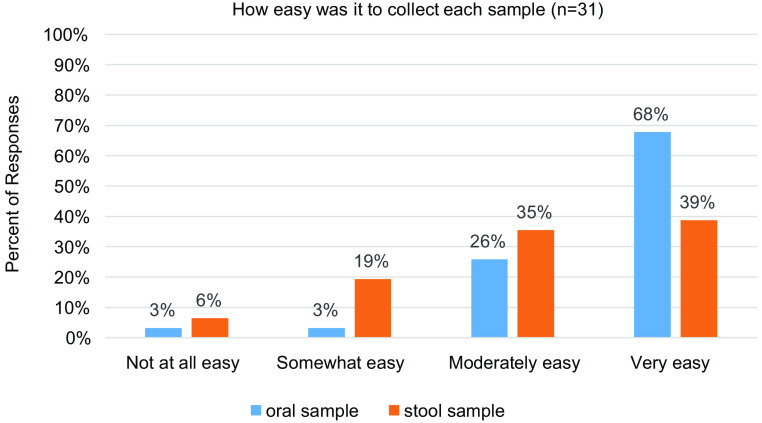
Ease of sample collection indicated by survey respondents.

While only 32% of survey respondents (*n* = 10) had previously participated in a telehealth visit prior to enrolling in this study, almost all were either very comfortable (74%) or moderately comfortable (19%) using technology such as smart phones or computers. Eighty-four percent of respondents found the telehealth platform to be “very easy” to use. Overall, survey participants felt the instructions on how to collect the samples were clear, but sample collection itself proved to be harder. Sixty-eight percent of respondents said collecting oropharyngeal samples was “very easy,” but only 39% of respondents said collecting the stool samples was “very easy” (Fig. 1).

We sought to understand if the remote nature of the trial affected the relationship between participants and research team members. Overall, survey respondents were very positive about their interactions with the research team, with 97% reporting they were very satisfied with how staff respected their privacy. Seventy-one percent of respondents said it was very easy to share any concerns they may have had with the study staff, and the remaining 29% said they had no concerns at all. Participants also said they were very confident (90%) in the skills and knowledge of the study staff and 97% responded that staff always treated them with courtesy and respect.

Although 97% (*n* = 30) of respondents had not previously participated in clinical research, 77% (*n* = 24) said they would consider taking part in another clinical research study in the future for a condition other than COVID-19. Of those who said they would consider participating in a future study, 96% (23/24) indicated they would be more likely to participate if the visits were not in-person. Fifty percent (12/24) were only “somewhat likely” to participate if a person had to come to their home for a health checkup or to collect samples (Table 2).

**Table 2.** Likelihood of survey participants engaging in future research endeavors

Question: How likely are you to participate if…	Not at all likely	Somewhat likely	Moderately likely	Very likely
…all study visits take place in person at a hospital or clinic?	29% (7/24)	50% (12/24)	17% (4/24)	4% (1/24)
…all study visits take place using a telehealth platform you can access from home?	0% (0/24)	4% (1/24)	17% (4/24)	79% (19/24)
…communication with the study staff always takes place over a smartphone, computer (PC), or telephone (landline)?	0% (0/24)	0% (0/24)	21% (5/24)	79% (19/24)
…you need to collect your own oral swab samples?	0% (0/24)	0% (0/24)	21% (5/24)	79% (19/24)
…someone needs to visit you in your home to do a health checkup or collect samples?	13% (3/24)	50% (12/24)	25% (6/24)	13% (3/24)
…you need to travel more than 30 minutes each way to take part in in-person study visits?	63% (15/24)	29% (7/24)	4% (1/24)	4% (1/24)

### Qualitative Interviews

Qualitative stakeholder interviews about their experiences with the telehealth trial and attitudes towards future remote trials were done with two trial participants and seven research team members. Despite the small number of participant interviews, we include the perspectives of these participants to provide depth to the survey findings.

#### Trial participant interviews

Feedback from interviews with the trial participants largely aligned with the survey findings. For instance, both participants shared that they thought telehealth visits were easy, short, and flexible and that they enjoyed interacting with trial staff. They also stated that they would have been less likely to participate in an in-person trial, citing barriers including travel time, feeling too ill to travel, and the potential for missed work.

Participant interviews added perspective to the survey findings that remote visits were easy for trial participants. When asked whether there was any benefit to the remote conduct of study visits, one participant shared:

*I think it [remote participation from home] is more effective in that people probably won’t drop out as much, because it’s more doable. I think more people will say “yes” to doing it because it’s much easier, and just knowing that someone is going to be checking in with you even via video everyday… no matter where you are, no matter where they are, I think is really helpful.*

Participants also expressed appreciation for the frequent remote monitoring of their condition:

- *Feeling like I had someone looking over me at a time when I felt like I had no one watching me, it was good.*
- *Everyone I talked to was super nice and were genuinely checking in on me*.

Survey data suggested that most participants found the sample collection “very or moderately easy” and instructions for obtaining the sample “very or moderately clear.”

One participant shared:

*… [H]aving everything delivered to my door and picked up, and having all of the coordinating of the pickups and drop-offs and everything taken care of for me made it really easy. The instructions were clear … I would structure future studies really similarly.*

Regarding their overall experience in the remote trial, the other participant stated:

*I think it’s a very positive step in the right direction for research … I think it’s going to make it so much easier for people, or researchers, to get good samples and get the information they need when you make it easier for people.*

Regarding suggestions for future remote trials, one participant interviewee recommended sending electronic reminders to participants on the days when samples should be collected.

#### Research team member interviews

All seven members of the clinical trial research team were interviewed about their experience working on the remote trial. Team member roles included trial oversight, conduct, data management, and analysis. All members had experiences in similar roles on at least one previous study, but the use of telehealth in clinical research was new to them all. Only two team members had previous experience with remote collection of samples.

Research team members identified facilitators, benefits, and challenges to conducting the remote trial, as shown in Table 3. All members highlighted the importance of an effective team environment in helping them to fulfill their role. Team members mentioned several strengths that fostered this environment, including effective coordination of logistics, interdisciplinary expertise, professionalism, effective leadership, and a high level of motivation and capability. The top barrier identified by members (n = 3) involved technology issues with the telehealth visits, including poor internet connection or cell phone coverage. Researchers who identified this challenge said that it did not have a lasting impact on their ability to conduct the visits. The use of backup platforms helped to overcome this barrier; when one platform was not working, the researcher and participant would switch to a second platform.

**Table 3.** Facilitators and barriers identified by research team members

Category	Facilitators and benefits	Barriers/challenges
**Study design**	Patient monitoring and follow-up were effectively completed remotely Data collection could be conducted remotely Sample self-collection and shipping were feasible Study protocol (i.e., participant inclusion and exclusion criteria) allowed for recruitment of good candidates (i.e., patients likely able to complete study activities) Intervention could be self-administered Remote randomization and assignment Participants recruited from a larger geographic area	Study start-up challenges Obtaining medication not locally available, repurposing Shipping company pickup schedule required sample storage at home Sample shipping delays led to missing data Participantsʼ compliance with study activities, that is, dating samples incorrectly No in-person visits led to assessment and diagnosis challenges
**Technology and resources**	Telehealth enabled participants to be “seen” easily and facilitated building researcher and participant connection and rapport Fairly reliable technology for telehealth visits or backup telephone visits was available Courier and sample shipping services were available and reliable Medications could be delivered to the home Remote consent technology was available and reliable	Monitoring visits were disrupted due to poor internet connections, lack of cell phone coverage, and connection delays Telehealth platform designed for clinical care visits rather than research visits Hospital technology issues including poor cell phone coverage Participants requiring interpreter services were not able to be seen via video
**Research participant**	Research participants were willing and able to learn how to self-collect samples in their home Remote participation was convenient, saved costs of travel and parking, and avoided navigating the hospital Research participants expressed to staff that they liked that they were seen regularly by research clinicians Individuals needing to adhere to isolation guidelines, or hesitant to go to a hospital, could participate Courier pickup of sample in the home was convenient and feasible Flexible appointment scheduling helped participants working from home, providing childcare, or needing symptoms assessed outside of the visit schedule	Participants found the sample schedule confusing
**Research team**	Team characteristics fostered collaboration, coordination, and creativity to overcome technical and logistical challenges Staff could see participants without concerns about COVID-19 exposure Study visits were short and staff was able to work from home making visit scheduling easier Telehealth visits allowed staff to get to know participants in the participant’s home setting	Could not ensure adequate fecal sample collection

Researchers who met with participants using telehealth technology (n = 4) estimated that video was used in 85-90% of visits. They found video was helpful for reading facial expressions, observing symptoms, and creating a connection with research participants that more closely resembled in-person visits compared to non-video remote interactions. Team members estimated that in the 10–15% of interactions where video was not used, participants typically either had poor internet connection, were multitasking, or were using translation services on which video was not available. These researchers (n = 4) believed video was the best and most convenient method of communication for the trial given the prohibition on in-person study visits early in the pandemic.

All research team members interviewed (n = 7) had a positive outlook on the use of telehealth in clinical trials following the trial. Some noted that the use of telehealth was more convenient, protected them from infection, helped with participant retention, and could be used as a strategy to engage diverse populations in research. They also recognized, however, that some trial designs could not be implemented remotely. When it came to the COVID-19 therapeutic trial, most team members (n = 5) felt that the trial would have been more difficult, if not impossible, to conduct in-person.

Researchers shared three key successes and several recommendations for future research studies using telehealth, as shown in Table 4. First, the trial was a “proof of concept” demonstrating the feasibility of telehealth visits and participant sample self-collection. This finding aligns with survey and interview data collected from research participants. Second, they reported a strength of their approach was to brainstorm about possible challenges that arose during the study and to devise solutions. Third, team members emphasized the importance of having a motivated, collaborative, team to successfully carry out the research.

**Table 4.** Clinical trial research team member recommendations for future remote trials

Category	Recommendations
**Technology and resources**	Ensure research teams have adequate supporting infrastructure and trial technology Tailor telehealth platform to better support remote research visits Make improvements to remote consenting application Leverage telehealth to recruit participants from a wider geographic region Provide participants with needed technology (i.e., smartphone) Ensure participants know how to use the technology
**Research team**	Ensure participants acquire sufficient skills for proper sample collection, dose scheduling, and data collection at enrollment Consider using videos for participant sample collection training Employ tools and technology to enable monitoring of participant vital sign collection Implement electronic reminders or other methods to ensure timely participant sample collection Partner with local labs to support at-home sample collection

## Discussion

This mixed methods evaluation of the experiences and perceptions of research participants and research team members suggests that remote trial designs can result in positive participant and research team experiences and the successful execution of study activities. Responding to the requirements of conducting research in the midst of the COVID-19 pandemic, the research team designed and executed a fully remote clinical trial. This allowed participants to maintain quarantine and the research team members (and their healthcare facility) to avoid unnecessary exposure to patients with COVID-19. Although not all trials can be conducted in this way, in addition to situations such as for COVID-19, remote designs may be effective at recruiting and retaining sufficient numbers of geographically diverse participants.

Our mixed methods study describes both participant and research team perspectives regarding the conduct of a remote therapeutic clinical trial during the COVID-19 pandemic. The urgency to rapidly identify novel therapies led to implementation of innovative trial designs that protected study staff from direct exposure to outpatients with COVID-19, fostered trial participants’ adherence to quarantine guidelines, and preserved the use of limited personal protective equipment. Within the public health emergency of a COVID-19 pandemic was a unique opportunity to employ remote technology in the conduct of therapeutic clinical trials and evaluate remote trial feasibility. Social media advertisements and automated 24-hour a day self-enrollment facilitated recruitment in certain remote trials; however, researchers experienced barriers in obtaining complete and accurate follow-up data [31]. Despite challenges, remote clinical trials present an opportunity beyond the pandemic to enhance participant-friendly trial design, reducing the expense of in-person clinic visits, and supporting clinical trial participation in the comfort of one’s home or workplace [32]. Applying lessons learned during the COVID-19 emergency to expanding remote trial conduct represents a chance to shift current trial paradigms with opportunity for enhanced study participant recruitment and retention.

The experience of delivering a fully remote clinical trial and evaluating participant and study staff experiences has many implications for future research. First, with the increased use of telehealth visits for routine care, potential research participants may be more familiar and comfortable with this technology. Both researchers and participants acknowledged that access to the right technology and supporting infrastructure (i.e., adequate internet connection and cell phone coverage) and the knowledge about how to use this technology were essential. Adaptation of remote platforms to accommodate study-specific design and protocol activities including remote enrollment, virtual study visits, and electronic patient-reported data collection is key to ensuring accurate, robust measurement of research outcomes.

Fully embracing remote clinical trial conduct will require development and implementation of broadly available participant education and training technology, sample collection strategies, and physiologic monitoring. Use of wearable biosensor technology, expansion of standardized, study-specific adaptable online surveys, questionnaires, and forms for remote data collection is a fertile area for implementation science research. Employing easily accessible online videos and participant manuals to provide or reinforce instructions about study activities, such as sample collection or the use of new forms of technology, are particularly important to this future research. Increasing clinical trial participant engagement in remote data collection requires development and refinement of broadly adaptable technology. Inclusion of clinical trial activities that are straightforward, convenient, and easily adopted by diverse populations is imperative. A broader vision for remote trials includes the ability to engage patients in remote and rural locations using accessible technology that enables a full range of study activities and data collection [33]. Engaging rural and underserved communities who are often excluded from clinical trial participation due to geographic or socioeconomic barriers through use of email reminders, text messaging alerts, and/or automated phone calls is a priority. Increasing emphasis on participant- or patient-centered design of user interfaces, data collection tools, and the integration of diagnostic devices such as pulse oximetry, continuous glucose monitors, and wearables can support remote trial conduct and accurate measurement of study outcome measures. Designs should include input from patients to ensure they are respectful and responsive to patient needs while also meeting research requirements.

While there may be certain therapeutic trial activities that must be delivered in person, such as radiographic scans or intravenous chemotherapy, other activities such as clinical evaluation via telehealth deserve further exploration. It is essential that the research community continues to identify strategies to engage with research participants virtually. Several telehealth modalities hold potential for this inquiry. One example would be the use of synchronous real-time telephone or audio-video interaction, typically using a smartphone, tablet, or computer. Another could be use of peripheral medical equipment such as digital stethoscopes or ultrasound sensors used by a clinician physically with the patient, while another clinician conducts a remote evaluation. Yet another could be use of asynchronous technology using images or data collected at one point in time and responded to later [34]. Further exploration of electronic patient-reported outcomes and shared decision-making is warranted, as are remote visit communication barriers and the inability to perform a full clinical exam [35].

In expanding access to clinical research trials via remote platforms, we must ensure equity for all patients, including those of lower socioeconomic status who may rely on public hotspots or library computers for internet connectivity. These barriers must be addressed to ensure health equity for all patients seeking care. Use of telehealth during the COVID-19 crisis can serve as a model for continued use beyond the pandemic [1].

This study had several limitations. Results from the surveys and interviews are limited to trial participants who were recruited and consented remotely and were interested and able to engage in a remote trial. The median age of the trial participants who responded to the survey was 31 years. This is lower than the nationwide, median age of COVID-19 cases of 37 years at the time the trial was ongoing in July 2020, and 38 in August 2020 [36]. Therefore, our study may reflect that younger people were more comfortable with remote technology, telehealth visits, or self-sampling. Further study is needed with older adults as well as with special populations. Our results may not reflect those that declined to participate due to lack of access to or comfort with technology, given that inclusion criteria included willingness to comply with all study procedures and availability for the duration of the study.

Another limitation to our survey development process was the lack of validated surveys in assessing patient satisfaction with clinical research telehealth platforms. Also, our survey pilot testing was limited to five English-speaking individuals who identified as female. We may have failed to collect representative feedback during the pilot testing process.

The small number of trial participants interviewed for the study represents an important limitation. Recruitment of trial participants for interviews was limited because of both delays in initiating interview study recruitment and because the clinical trial ended before attaining the intended sample size when COVID-19 infection dropped precipitously in Massachusetts. All research team members invited to participate in the interview accepted. Future research might validate these findings by collecting similar data from multiple research teams conducting remote trials. Despite the limited number of participants, interview participants’ responses reflected and gave additional voice to the findings from the survey. Together with the feedback elicited in staff interviews, our data supported the conclusion that remote clinical trial conduct and participation was feasible, desirable, and successful in completing study activities. Additional research is needed to confirm these findings including in non-COVID-19 treatment trials and more diverse participant populations.

Remote clinical research holds promise for engaging more, and more diverse, patients in clinical studies. While this design may allow researchers to conduct studies more efficiently, an even more important benefit may be the broader applicability of the research results to the patient population. Wider application and development seem warranted.
